# The Efficacy and Safety of Selective H_1_-Antihistamine versus Leukotriene Receptor Antagonist for Seasonal Allergic Rhinitis: A Meta-Analysis

**DOI:** 10.1371/journal.pone.0112815

**Published:** 2014-11-10

**Authors:** Yu Xu, Jixiang Zhang, Jun Wang

**Affiliations:** 1 Department of Otolaryngology, Renmin Hospital of Wuhan University, Wuhan 430060, Hubei Province, China; 2 Department of Gastroenterology, Renmin Hospital of Wuhan University, Wuhan 430060, Hubei Province, China; University of Southampton School of Medicine, United Kingdom

## Abstract

**Background:**

Both selective H_1_-antihistamine (SAH) and leukotriene receptor antagonist (LTRA) have been shown to be effective in treating patients with seasonal allergic rhinitis (SAR), but it is still uncertain which treatment option is optimal. This meta-analysis was aimed to compare the efficacy and safety of SAH and LTRA for SAR.

**Materials and Methods:**

PubMed, EMBASE and the Cochrane Library were searched for all eligible studies that compared the efficacy and safety of SAH and LTRA for SAR up to September 7, 2014. The pooled mean difference (MD), odd ratios (ORs) and 95% confidence intervals (95% CIs) were calculated using a fixed- or random-effects model.

**Results:**

Nine studies with 5781 SAR patients were included. The results showed that SAH is superior to LTRA in terms of the daytime eye symptoms score (DESS) and composite symptoms score (CSS) for SAR (MD = 0.06, 95% CI, 0.03 to 0.10, *P* = 0.000, *I* ^2^ = 99%; MD = 0.03, 95% CI, 0.01 to 0.05, *P* = 0.010, *I* ^2^ = 98%), whereas LTRA overmatched SAH with respect to the night-time symptoms score (NSS) (MD = −0.04, 95% CI, −0.05 to −0.02, *P* = 0.000, *I* ^2^ = 97%). Additionally, the results of subgroup analysis indicated that the dose, duration and gender of the patients might impact the comparisons of the effects of SAH and LTRA on their efficacy for SAR.

**Conclusion:**

This meta-analysis suggested that SAH and LTRA have similar effects and safety for SAR, but SAH is more appropriate for daytime nasal symptoms (congestion, rhinorrhea, pruritus and sneezing), while LTRA is better suited for nighttime symptoms (difficulty going to sleep, nighttime awakenings, and nasal congestion on awakening), respectively. Meanwhile, the dose, duration and gender of patients may influence the anti-SAR effects of SAH and LTRA.

## Introduction

Seasonal allergic rhinitis (SAR), also known as hay fever, whose symptoms are complex and characterized by rhinorrhea, nasal congestion, sneezing and nasopharyngeal itching, is an inflammatory condition of the upper airways that occurs in response to airborne allergen (typically tree, grass and weed pollens) exposure in sensitized individuals [1], [2]. SAR results from the binding of an inhaled aeroallergen to immunoglobulin E (IgE) on the surface of mast cells in the nasal mucosa [3]. Compared to perennial allergic rhinitis (PAR), which accounts for approximately 40% of allergic rhinitis (AR), SAR affects between 30 and 40% of adults and children, and the prevalence is increasing [4]. To date, the treatments for allergic rhinitis include allergen avoidance, pharmacotherapy, and immunotherapy. However, for SAR, total allergen avoidance may be undesirable because it may require limiting the time spent outdoors. Therefore, pharmacotherapy, including selective H_1_-antihistamine (SAH), corticosteroids, decongestants, bronchodilators, intranasal mast cell stabilizers and leukotriene receptor antagonists (LTRA), is preferable to allergen avoidance for the symptom relief of SAR [5]–[7]. Among these drugs, SAH and LTRA have attracted increasing attention.

The mechanism of LTRA in the treatment of SAR involves blocking the cysteinyl leukotriene receptor. Cysteinyl leukotrienes, including leukotriene LTC4, LTD4, and LTE4, which are peptide-conjugated lipids produced by activated basophils, eosinophils, mast cells and macrophages [8], are important mediators of airway inflammation that may also be important in the pathogenesis of allergic rhinitis. Nasal antigen challenge in sensitized individuals leads to high levels of cysteinyl leukotrienes in nasal lavage fluids, whereas nasal challenge with cysteinyl leukotrienes produces nasal obstruction and rhinorrhea [9]–[11]. Additionally, SAH has long been maintaining a crucial position among the agents used in the pharmacological management of SAR. Histamine, produced from L-histidine through the action of histidine decarboxylase and stored in mast cells and basophils in the nasal mucosa, plays an important role in the immunoregulation of allergic inflammation [12]. There are 4 subtypes of histamine receptors, including histamine 1 receptor (H1R), H2R, H3R and H4R. Mainly located on vascular smooth muscle cells and endothelial cells, H1R mediates the histamine-associated vasopermeability and vasodilatation, which are important to the emergence of symptoms of rhinorrhea and congestion [13]. Therefore, antihistamine, especially SAH, may help to alleviate histamine 1 receptor-associated redness, itching, swelling, rhinorrhea, and conjunctivitis [14], [15].

Because both of SAH and LTRA can treat SAR, choosing the one that is optimal according to different symptoms and symptom severity remains a challenge for rhinologists. Recently, a number of studies have compared the efficacy and safety of SAH with LTRA for SAR, but the results remain inconclusive. Therefore, to overcome the limitations of these individual studies and obtain a standard to guide the use of these two medicines, we conducted a meta-analysis of the previously published studies, comparing the efficacy and safety of SAH with LTRA for SAR.

## Materials and Methods

### Search strategy

Two reviewers independently reviewed PubMed, EMBASE and the Cochrane Library up to September 7, 2014. All eligible studies that compared the efficacy and safety of SAH and LTRA for SAR were included. Relevant studies were identified using the following key words and subject terms: ‘‘seasonal allergic rhinitis’’ or ‘‘SAR’’, ‘‘selective H_1_-antihistamine’’ or ‘‘loratadine’’ or “desloratadine” or “acrivastine” or “fexofenadine” or “levocetirizine”, and ‘‘leukotriene receptor antagonist’’ or ‘‘LTRA’’ or ‘‘montelukast’’ or “zafirlukast” or “pranlukast”. A hand search of the reference lists in the related studies was also performed. The search was limited to humans and without language limitations.

### Inclusion and Exclusion Criteria

Studies were included into the meta-analysis if they met the following criteria: (1) randomized controlled trials or case-control studies; (2) compared the efficacy and safety of SAH with LTRA for SAR; (3) had detailed information on cases and controls; and (4) had specific evaluating standards. Studies were excluded if they met one of the following conditions: (1) did not compare the efficacy and safety of SAH with LTRA; (2) perennial allergic rhinitis; (3) case-only studies, case reports and review articles; (4) lacked sufficient information, and (5) lacked a control group.

### Data Extraction

Independently, two investigators extracted data, including the first author, year of publication, country, study duration, age, gender, medication duration, drug dose, treatment methods and primary endpoints. Any encountered discrepancies were resolved by consensus.

### Endpoints

The daytime nasal symptoms score (DNSS), which was defined as the mean of four daytime nasal symptoms (congestion, rhinorrhea, pruritus and sneezing), was treated as the primary endpoint. Additionally, the daytime eye symptoms score (DESS, mean of scores for tearing, pruritus, redness and puffiness), composite symptoms score (CSS, mean of daytime nasal and nighttime symptoms scores), nighttime symptoms score (NSS, mean of scores for difficulty going to sleep, nighttime awakenings, and nasal congestion on awakening), rhinoconjunctivitis quality-of-life scores (RQOLS, mean of scores for activity, sleep, nasal symptoms, ocular symptoms, nonnose/non-eye symptoms, practical problems, and emotions) and safety were treated as the secondary endpoints.

### Statistical Analysis

Meta-analysis was performed using the Cochrane Collaboration RevMan 5.1 and STATA package version 12.0 (Stata Corporation, College Station, Texas). The pooled mean difference (MD), odd ratios (ORs) and 95% confidence intervals (95% CIs) were calculated to compare the efficacy and safety of SAH with LTRA for SAR. Additionally, subgroup analyses were performed based on the gender, treatment duration and dose when adequate data were available. A χ^2^-test-based Q statistic was performed to assess the between-study heterogeneity. When *I* ^2^>50% and *P*<0.1, heterogeneity was considered statistically significant, and a random effects model was used to analyze the data. When that was not the case, a fixed effects model was chosen. The Egger’s test was used to assess the publication bias.

Sensitivity analysis was performed to determine the studies that obviously influence the result.

## Results

### Studies Included in the Meta-analysis

**Figure 1:**
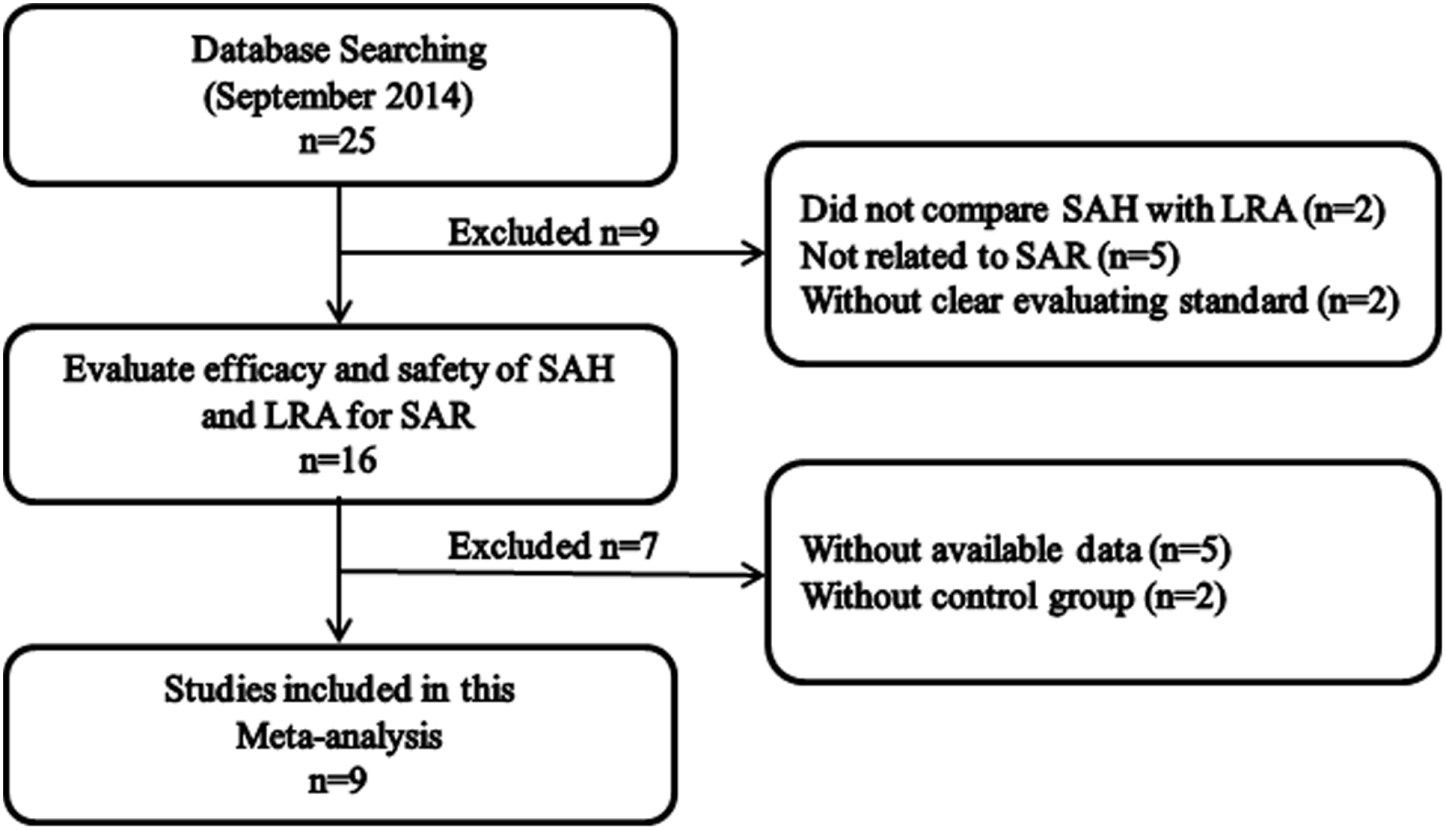
The screening process of studies.

According to the searching strategy, 25 potentially relevant studies were chosen. Based on the inclusion criteria and criteria, 9 studies [16]–[24] were included into this meta-analysis and 16 were excluded (Fig. 1). Eight studies [16]–[20], [22]–[24] compared the efficacy of SAH and LTRA for SAR. Among these studies, six [16]–[20], [22] with respect to the DNSS, five [16]–[19], [22] on the DESS, seven [16]–[20], [22], [23] on the CSS, six [16]–[19], [22], [24] reporting on the NSS and four [17]–[19], [22] examined the RQOLS. Meanwhile, seven studies [16]–[19], [21]–[23] examined the adverse events for SAH and LTRA (Table 1).

**Table 1:** Characteristics of Studies Included in the Meta-analysis.

First author	Year	Treatment	Dose(mg)	Duration(weeks)	N	Age	Gender(M/F)	Race(white%)	HistoryAR (y)	Historyof asthma(%)	Historyof AC(%)	DNSS	DESS	CSS	NSS	RQLQ	Adverseevents (%)
Meltzer [16]	2000	montelukast	10	2	95	33.0	40/55	–	18±13	26.3	93.7	−0.36(−0.47,−0.26)	−0.28(−0.40,−0.15)	−0.39(−0.48,−0.30)	−0.29(−0.39,−0.19)	–	7.4
		montelukast	20	2	90	34.5	33/57	–	18±12	32.3	95.6	−0.29(−0.39,−0.18)	−0.14(−0.27,−0.02)	−0.31(−0.41,−0.22)	−0.21(−0.31,−0.10)	–	10
		loratadine	10	2	92	34.5	43/49	–	19±13	35.9	94.6	−0.34(−0.44,−0.23)	−0.25(−0.37,−0.12)	−0.32(−0.41,−0.22)	−0.19(−0.30,−0.09)	–	9.8
Philip [17]	2002	montelukast	10	2	348	37	233/115	83	18±12	27	88	−0.13(−0.21,−0.06)	−0.14(−0.22,−0.06)	−0.13(−0.20,−0.07)	−0.14(−0.20,−0.07)	−0.89(−1.01,−0.77)	19
		loratadine	10	2	602	36	390/212	84	18±12	25	87	−0.24(−0.31,−0.17)	−0.20(−0.28,−0.13)	−0.17(−0.24,−0.11)	−0.09(−0.15,−0.03)	−0.99(−1.08,−0.90)	21
van Adelsberg (A) [18]	2003	montelukast	10	2	522	36	147/301	82	19±12	22	89	−0.33(−0.37,−0.28)	−0.28(−0.33,−0.23)	−0.30(−0.35,−0.26)	−0.28(−0.32,−0.23)	−0.85(−0.94,−0.75)	–
		loratadine	10	2	171	39	61/119	81	21±13	24	90	−0.45(−0.52,−0.37)	−0.33(−0.41,−0.26)	−0.36(−0.43,−0.30)	−0.25(−0.32,−0.19)	−0.85(−1.00,−0.70)	–
		montelukast	10	4	522	36	147/301	82	19±12	22	89	−0.43(−0.48,−0.38)	−0.37(−0.42,−0.31)	−0.40(−0.45,−0.36)	−0.36(−0.40,−0.32)	−0.13(−0.21,−0.06)	2.5
		loratadine	10	4	171	39	61/119	81	21±13	24	90	−0.50(−0.58,−0.42)	−0.39(−0.47,−0.31)	−0.42(−0.49,−0.36)	−0.32(−0.39,−0.25)	−1.08(−1.25,−0.91)	0.6
van Adelsberg (B) [19]	2003	montelukast	10	2	522	36	196/326	82	17±12	23	89	−0.38(−0.43,−0.33)	−0.28(−0.32,−0.23)	−0.34(−0.38,−0.30)	−0.28(−0.32,−0.24)	−0.90(−1.00,−0.81)	17
		loratadine	10	2	171	35	71/100	82	18±12	26	89	−0.47(−0.55,−0.39)	−0.40(−0.47,−0.32)	−0.39(−0.46,−0.32)	−0.28(−0.35,−0.21)	−0.98(−1.15,−0.81)	15
Lu (study1) [20]	2009	montelukast	10	2	112	35.6	42/70	79.5	19±11	22.3	88.4	−0.36(−0.46,−0.25)	–	−0.31(−0.40,−0.21)	–	–	–
		loratadine	10	2	116	34.8	41/75	79.3	18±11	27.6	91.4	−0.53(−0.63,−0.42)	–	−0.42(−0.52,−0.33)	–	–	–
Lu (study2) [20]	2009	montelukast	10	2	103	31.1	39/64	89.3	19±11	–	72.8	−0.34(−0.44,−0.24)	–	−0.39(−0.50,−0.27)	–	–	–
		loratadine	10	2	164	30.6	65/99	89.0	17±10	–	78.0	−0.29(−0.37,−0.20)	–	−0.40(−0.49,−0.30)	–	–	–
Baena-Cagnani [21]	2003	montelukast	10	4	311	33.7	122/189	79	–	–	–	–	–	–	–	–	11.9
		Desloratadine	5	4	311	32.3	116/195	82	–	–	–	–	–	–	–	–	15.8
Nayak [22]	2002	montelukast	10	2	155	37	102/53	83	18±13	18	93	−0.23(−0.35,−0.11)	−0.20(−0.32,−0.08)	−0.20(−0.31,−0.10)	−0.17(−0.28,−0.07)	−1.09(−1.26,−0.92)	17
		loratadine	10	2	301	37	191/110	80	19±13	22	89	−0.26(−0.37,−0.16)	−0.23(−0.34,−0.13)	−0.21(−0.30,−0.12)	−0.14(−0.23,−0.05)	−1.06(−1.19,−1.93)	17
Patel [23]	2008	montelukast	10	2 days	149	34.6	63/86	78	–	–	–	–	–	−5.90(−19.5,−7.7)	–	–	12.8
		Levocetirizine	5	2 days	152	38.6	65/87	83	–	–	–	–	–	−8.40(−22.0,−5.8)	–	–	15.8
Michele (study1) [24]	2000	montelukast	10	2	138	–	–	–	–	–	–	–	–	–	−0.21(−0.36,−0.06)	–	–
		loratadine	10	2	145	–	–	–	–	–	–	–	–	–	−0.26(−0.41,−0.11)	–	–
Michele (study2) [24]	2000	montelukast	10	2	158	–	–	–	–	–	–	–	–	–	−0.08(−0.23,−0.06)	–	–
		loratadine	10	2	160	–	–	–	–	–	–	–	–	–	−0.00(−0.13,0.14)	–	–

AR, allergic rhinitis;AC, allergic conjunctivitis; DNSS, daytime nasal symptoms score; DESS, daytime eye symptoms score; CSS, composite symptoms score; NSS, nighttime symptoms score; RQLQ, rhinoconjunctivitis quality-of-life scores.

### The efficacy of Selective H1-antihistamine and Leukotriene Receptor antagonist for Seasonal allergic rhinitis

A summary of the meta-analysis findings on the efficacy of Selective H1-antihistamine and Leukotriene Receptor antagonist for seasonal allergic rhinitis is shown in Tables 2 and 3.

**Table 2:** Pooled Analysis for the Effective and Safety of Leukotriene antagonist versus Selective H_1_ antihistamine for Seasonal allergic rhinitis.

Outcomes	comparison (n)	Patients		Test of Effective and Safety		Test of Heterogeneity		Publication Bias *P*-value
		LTRA	SAH	MD/OR (95% CI)	*P*-value	*P*-value	*I* ^2^ (%)	
DNSS	9	2469	1880	0.06(0.03, 0.10)	0.000	0.000	99	0.997
DESS	7	2254	1600	0.04(−0.01, 0.08)	0.090	0.000	99	0.547
CSS	10	2618	2032	0.03(0.01, 0.05)	0.010	0.000	98	0.426
NSS	9	2023	1734	−0.04(−0.05, −0.02)	0.000	0.000	97	0.605
RQOLS	5	2069	1416	0.22(−0.10, 0.54)	0.180	0.000	100	0.132
Adverse events	8	2192	1892	0.92(0.76, 1.11)	0.380	0.690	0	0.304

LTRA, Leukotriene antagonist; SAH, Selective antihistamine; DNSS, Daytime nasal symptoms score; DESS, Daytime eye symptoms score; CSS, Composite symptoms score; NSS, Night-time symptoms score; RQOLS, Rhinoconjunctivitis quality-of-Life score.

**Table 3:** Subgroup Analysis for the Effective and Safety of Leukotriene antagonist versus Selective H_1_ antihistamine for Seasonal allergic rhinitis.

Comparison	Subgroup	Test of Association		Test of Heterogeneity		Subgroup difference	
		OR (95% CI)	*P*-value	*P*-value	*I* ^2^ (%)	*P*-value	*I* ^2^ (%)
DNSS							
Dose of LTRA	10 mg	0.07(0.03,0.10)	0.000	0.000	99	0.440	0
	20 mg	0.05(0.04,0.06)	0.000	–	–		
Duration	2 weeks	0.06(0.02,0.10)	0.002	0.000	99	0.720	0
	4 weeks	0.07(0.06,0.08)	0.000	–	–		
Gender	Female >50%	0.06(0.02,0.11)	0.006	0.000	99	0.850	0
	Male >50%	0.07(−0.01,0.15)	0.080	0.000	99		
DESS							
Dose of LTRA	10 mg	0.02(−0.02,0.07)	0.310	0.000	99	0.000	91.6
	20 mg	0.11(0.09,0.13)	0.000	–	–		
Duration	2 weeks	0.04(−0.01,0.09)	0.120	0.000	99	0.460	0
	4 weeks	0.02(0.01,0.03)	0.000	–	–		
Gender	Female >50%	0.03(−0.04,0.10)	0.360	0.000	100	0.720	0
	Male >50%	0.05(0.02,0.07)	0.002	0.000	95		
CSS							
Dose of LTRA	10 mg	0.03(0.01,0.05)	0.010	0.000	99	0.150	50.8
	20 mg	0.01(0.00,0.02)	0.160	–	–		
SAH	loratadine	0.03(0.00,0.05)	0.020	0.000	98	0.520	0
	Levocetirizine	0.02(0.01,0.03)	0.000	–	–		
Duration	2 weeks	0.03(0.00,0.05)	0.020	0.000	98	0.520	0
	4 weeks	0.02(0.01,0.03)	0.010	–	–		
Gender	Female >50%	0.03(0.00,0.06)	0.060	0.000	99	0.890	0
	Male >50%	0.03(0.00,0.05)	0.090	0.000	96		
NSS							
Dose of LTRA	10 mg	−0.03(−0.07,0.01)	0.100	0.000	100	0.620	0
	20 mg	−0.02(−0.04,0.00)	0.010	–	–		
Duration	2 weeks	−0.03(−0.07,0.01)	0.150	0.000	100	0.700	0
	4 weeks	−0.04(−0.15,0.03)	0.000	–	–		
Gender	Female >50%	−0.04(−0.06,−0.01)	0.004	0.000	97	0.820	0
	Male >50%	−0.04(−0.06,−0.02)	0.000	0.000	92		
RQOLS							
Duration	2 weeks	−0.04(−0.02,0.10)	0.230	0.000	99	0.000	99.9
	4 weeks	0.95(0.94,0.96)	0.000	–	–		
Gender	Female >50%	0.34(−0.25,0.93)	0.260	0.000	100	0.320	0
	Male >50%	0.04(−0.09,0.16)	0.590	–	–		
Adverse enents							
Dose of LTRA	10 mg VS. 10 mg	0.99(0.79,1.24)	0.920	0.530	0	0.430	0
	10 mg VS. 5 mg	0.74(0.51,1.08)	0.120	0.850	0		
	20 mg VS. 10 mg	1.02(0.39,2.71)	0.960	–	–		
SAH	loratadine	0.99(0.79,1.24)	0.940	0.680	0	0.190	41.4
	Levocetirizine	0.74(0.51,1.08)	0.120	0.850	0		
Gender	Female >50%	0.92(0.71,1.20)	0.540	0.470	0	0.960	0
	Male >50%	0.91(0.69,1.21)	0.520	0.720	0		

LTRA, Leukotriene antagonist; SAH, Selective antihistamine; DNSS, Daytime nasal symptoms score; DESS, Daytime eye symptoms score; CSS, Composite symptoms score; NSS, Night-time symptoms score; RQOLS, Rhinoconjunctivitis quality-of-Life score.

**Figure 2:**
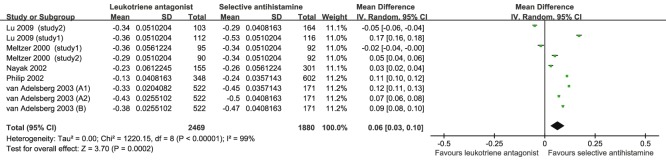
Pooled Analysis for DNSS of leukotriene antagonist versus selective H_1_ antihistamine for Seasonal allergic rhinitis.

Six studies [16]–[20], [22] with 9 comparisons, which included a total of 2469 patients who orally took LTRA and 1880 who took SAH, compared the DNSS of SAH and LTRA for SAR. SAH was better than LTRA according to the DNSS for SAR (MD = 0.06, 95% CI, 0.03 to 0.10, *P* = 0.000, *I* ^2^ = 99%) (Table 2 and Fig. 2). According to the subgroup analysis by the dose of LTRA, the DNSS of SAH and LTRA are similar for both 10 and 20 mg of LTRA. Additionally, on the basis of subgroup analysis by the treatment duration, there was no significant difference between SAH and LTRA for both 2 and 4 weeks of treatment. Additionally, according to the subgroup analysis by gender, when female >50%, SAH performed better than LTRA according to the DNSS, whereas, when male >50%, SAH is similar to LTRA.

Five studies [16]–[19], [22] with 7 comparisons, including a total of 2254 patients who took LTRA orally and 1600 who took SAH, compared the DESS of SAH and LTRA for SAR. There was no significant difference between SAH and LTRA on the DESS for SAR (MD = 0.04, 95% CI, −0.01 to 0.08, *P* = 0.090, *I* ^2^ = 99%) (Table 2 and Fig. S1). According to the subgroup analysis by the dose of LTRA, the DESS of SAH and LTRA are similar. However, on the basis of subgroup analysis according to the treatment duration, SAH is superior to LTRA when the duration is 4 weeks but not 2 weeks. Furthermore, the results of the subgroup analysis by gender showed that, when male >50%, SAH performed better than LTRA, whereas, when female >50%, SAH is similar to LTRA.

**Figure 3:**
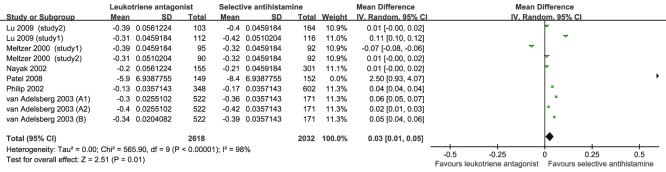
Pooled Analysis for CSS of leukotriene antagonist versus selective H_1_ antihistamine for Seasonal allergic rhinitis.

Seven studies [16]–[20], [22], [23] with 10 comparisons, involving 2618 patients who took LTRA orally and 2032 who took SAH, compared the CSS of SAH and LTRA for SAR. SAH performed better than LTRA for the CSS for SAR (MD = 0.03, 95% CI, 0.01 to 0.05, *P* = 0.010, *I* ^2^ = 98%) (Table 2 and Fig. 3). Based on the subgroup analysis by the dose of LTRA, 10 mg but not 20 mg of SAH, performed better than LTRA. Additionally, according to the subgroup analysis by SAH, both loratadine and levocetirizine advanced over LTRA. On the basis of subgroup analysis by treatment duration, SAH is superior to LTRA when the duration is 4 and 2 weeks. Moreover, the results of subgroup analysis by gender showed that, irrespective of whether male >50% or female >50%, SAH performed better than LTRA.

**Figure 4:**
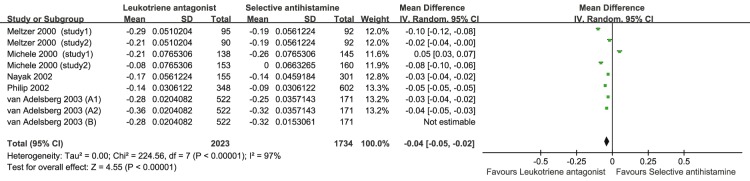
Pooled Analysis for NSS of leukotriene antagonist versus selective H_1_ antihistamine for Seasonal allergic rhinitis.

Six studies [16]–[19], [22], [24] with 9 comparisons, including 2023 patients who took LTRA orally and 1734 who took SAH, compared the NSS of SAH and LTRA for SAR. LTRA performed better than SAH with respect to the NSS for SAR (MD = −0.04, 95% CI, −0.05 to −0.02, *P* = 0.000, *I* ^2^ = 97%) (Table 2 and Fig. 4). According to the subgroup analysis by the dose of LTRA, 20 mg but not 10 mg of SAH performed better than LTRA. Furthermore, on the basis of subgroup analysis by treatment duration, SAH is superior to LTRA when the duration is 4 but not 2 weeks. The subgroup analysis by gender showed that for both male >50% and female >50%, SAH performed better than LTRA.

Four [17]–[19], [22] with 5 comparisons, including 2069 patients who took LTRA orally and 1416 who took SAH, compared the RQOLS of SAH and LTRA for SAR. There was no significant difference between SAH and LTRA for SAR (MD = 0.22, 95% CI, −0.10 to 0.54, *P* = 0.180, *I* ^2^ = 100%) (Table 2 and Fig S2). On the basis of subgroup analysis by treatment duration, SAH is superior to LTRA when the duration is 4 but not 2 weeks. However, the subgroup analysis by gender showed that SAH was similar to LTRA for both male >50% and female >50%.

### The Safety of Selective H1-antihistamine and Leukotriene Receptor antagonist for Seasonal allergic rhinitis

A summary of the meta-analysis findings involving the safety of Selective H1-antihistamine and Leukotriene Receptor antagonist for seasonal allergic rhinitis is shown in Tables 2 and 3.

Seven studies [16]–[19], [21]–[23] with 8 comparisons, consisting of 2192 patients who took LTRA orally and 1892 who took SAH, examined the adverse events of SAH and LTRA. There was no significant difference between SAH and LTRA with respect to adverse events (MD = −0.92, 95% CI, 0.76 to 1.11, *P* = 0.380, *I* ^2^ = 0%) (Table 2 and Fig S3).

### Test of Heterogeneity and Publication Bias

Heterogeneity of the included studies with respect to each outcome is presented in Table 2. Significant heterogeneity was found in most analyses except for adverse events. Though we have tried our best to determine the sources of heterogeneity by subgroup analysis, no obvious sources of heterogeneity were found.

The Egger’s test was used to assess the publication bias. According to the Egger’s Test, there was no evidence of publication bias for any of the analyses (Table 2).

### Sensitivity analysis

According to the results of the sensitivity analysis, none of the studies had a significant impact on the pooled analysis.

## Discussion

The first major finding of this meta-analysis was that, according to the DNSS, SAH treated SAR better than LTRA. In other words, compared with LTRA, SAH is more appropriate for patients with SAR whose cardinal symptoms are daytime nasal congestion, rhinorrhea, pruritus and sneezing. This could be explained by the following reasons: firstly, the cardinal symptom during daytime of SAR is rhinorrhea, sneezing, and pruritus, however nasal congestion, which contributes to reduced sleep quality and daytime somnolence, is not the most highlighted symptom during daytime but is the most important symptom during nighttime [25], [26]. Meanwhile, SAH has been demonstrated to be able to relieve nasal symptoms of early phase such as rhinorrhea, sneezing, and pruritus but have less effect on nasal congestion, whereas LTRA contributes to the remission of nasal congestion which is the late phase reaction by increasing vascular permeability, tissue edema, and mucus secretion, and are involved in inflammatory cell recruitment [27]–[29]. So, SAH is more appropriate for daytime nasal symptoms, while LTRA is better suited for nighttime symptoms. Additionally, another possibility is that the time course of being taken and release of LTRA differs from that of SAH in allergic rhinitis (montelukast often being taken at bedtime and rapidly absorbed achieving peak plasma concentration in 3 to 4 hours and effective throughout the 24-hour treatment period), thus leading to a dissimilar time course of response to therapy [18], [30], [31]. Meanwhile, the results of our meta-analysis showed that the superiority of SAH may disappear in females and the reasons for this are unclear. The possible explanation is that most SAH undergo hepatic metabolism via the (cytochrome P450 system, CYP) and gender differences have been described in the activity of CYP (CYP2D6, CYP3A4, CYP1A1/2 and CYP2C19) [32], [33], which may influence the efficacy of SAH and LTRA for SAR patients of different gender, but this supposition should be confirmed by more studies. Additionally, the dose (10 mg or 20 mg) and duration (2 weeks or 4 weeks) did not significantly influence the results.

Meanwhile, the second finding of our meta-analysis was that SAH was superior to LTRA for treating SAR according to the CSS, which means that SAH may be a better choice for SAR patients who have both daytime nasal (nasal congestion, rhinorrhea, pruritus and sneezing by day) and nighttime symptoms (difficulty going to sleep, nighttime awakenings, and nasal congestion on awakening). However, the subgroup analysis showed the type of SAH, duration and gender did not impact the results of pooled analysis. Interestingly, the different doses of SAH may influence the results; 10 or 20 mg SAH is more effective than LTRA according to the CSS. A high level of SAH may decrease the release of histamine in excess, promoting the positive modulation of histamine on sympathetic neurotransmission and resulting in the increased release of histamine. However, this explanation also should be demonstrated in a series of studies.

Furthermore, the third major finding was that LTRA performed better than SAH according to the NSS (difficulty going to sleep, nighttime awakenings, and nasal congestion on awakening) for SAR. The possible explanation is SAH is more appropriate for daytime nasal symptoms, while LTRA is more appropriate for nighttime symptoms. Additionally, the increase in the dose and treatment duration might contribute to the further superiority of LTRA.

The fourth finding of our meta-analysis was that, in terms of the DESS (tearing, pruritus, redness and puffiness during the daytime) and RQOLS (mean of scores for activity, sleep, nasal symptoms, ocular symptoms, nonnose/non-eye symptoms, practical problems, and emotions), SAH and LTRA have a similar effect. However, in males, SAH performed better than LTRA in terms of DESS, which also could be explained by the different superiority of SAH and LTRA for SAR and gender differences in the activity of CYP. However, the dose, gender of the patients and duration of treatment did not influence the results of the comparisons with respect to the DESS and RQOLS.

Additionally, the adverse reactions of SAH and LTRA are the critical problems that could not be ignored. Due to the different distribution of histamine and cysteinyl leukotrienes receptors, the adverse reactions of SAH and LTRA are different. H1R is expressed in neurons, airway and vascular smooth muscle cells, hepatocytes, chondrocytes, endothelial cells and inflammatory cells. Therefore, the adverse reactions of SAH include a central nervous system response (sedation, lethargy and fatigue), gastrointestinal reaction (thirst, anorexia, constipation and diarrhea), granulocytopenia and hemolytic anemia [34]. Meanwhile, leukotrienes receptors, which are mainly distributed in airway and vascular smooth muscle cells, monocytes and macrophages may cause adverse effects of different severity, including headache, gastrointestinal disturbances, pharyngitis, upper respiratory tract infection and rash [35]. According to the results of our meta-analysis, the adverse rate of LTRA is 13.8%, whereas that of SAH is 18.5%, but there is no significant difference between them. Additionally, the type of SAH, dose of SAH and LTRA, duration and gender did not impact the results of pooled analysis.

The combination of these agents has a reportedly higher improvement compared to monotherapy with either alone [16], [36]. In our study, SAH was more appropriate for patients with SAR whose cardinal symptoms are daytime nasal congestion, rhinorrhea, pruritus and sneezing and LTRA performed better for patients whose cardinal symptoms include difficulty going to sleep, nighttime awakenings, and nasal congestion on awakening. Therefore, the different aspects of the superiority of SAH and LTRA may indirectly support the combination of SAH and LTRA for SAR, but that should be verified by multicenter, random double-blind, controlled trials.

There are three limitations of this meta-analysis should be addressed. First, most pooled analyses have significant heterogeneity, excluding adverse events. Though tried to determine the sources of heterogeneity by subgroup analysis, no obvious sources of heterogeneity were found. Additionally, none of the studies dramatically influenced the results of pooled analysis according to sensitivity analysis, confirming the stability of our results. Second, several relevant studies have been excluded because of incomplete raw data. Third, because substantial important information could not be obtained from most included studies, relevant stratifications could not be performed for many studies.

In conclusion, our meta-analysis suggested that SAH and LTRA have similar effects and safety for SAR, but SAH is more appropriate for daytime nasal symptoms, while LTRA is more appropriate for nighttime symptoms. Meanwhile, the dose, duration and gender of patients may influence the anti-SAR effects of SAH and LTRA. Additionally, genetic and environmental factors should be investigated in the future.

## Supporting Information

Figure S1 **Pooled Analysis for DESS of leukotriene antagonist versus selective H1 antihistamine for Seasonal allergic rhinitis.**
(EPS)

Figure S2 **Pooled Analysis for RQOLS of leukotriene antagonist versus selective H1 antihistamine for Seasonal allergic rhinitis.**
(EPS)

Figure S3 **Pooled Analysis for adverse events of leukotriene antagonist versus selective H1 antihistamine for Seasonal allergic rhinitis.**
(EPS)

Checklist S1 **PRISMA Checklist.**
(DOC)

Checklist S2 **MOOSE Checklist.**
(DOC)
