# Supplementary material for: The Efficacy and Safety of Selective H1-Antihistamine versus Leukotriene Receptor Antagonist for Seasonal Allergic Rhinitis: A Meta-Analysis
Source: PLoS One. 2014 Nov 10;9(11):e112815. doi: 10.1371/journal.pone.0112815 (PMC4226613; doi:10.1371/journal.pone.0112815)
Supplement: Checklist S2 — MOOSE Checklist. (DOC) [file pone.0112815.s005.doc]

**MOOSE Checklist**

**The efficacy and safety of Selective H1-antihistamine versus Leukotriene Receptor antagonist for Seasonal allergic rhinitis: A Meta-analysis**

Yu Xu1*, Ji-Xiang Zhang

1 Department of Otolaryngology, Renmin Hospital of Wuhan University, Wuhan 430060, Hubei Province, China

2 Department of Gastroenterology, Renmin Hospital of Wuhan University, Wuhan 430060, Hubei Province, China

Yu Xu and Ji-Xiang Zhang contributed equally to this work.

*Corresponding author: Yu Xu, MD, PhD, Department of Otolaryngology, Renmin Hospital of Wuhan University, Wuhan 430060, Hubei Province, China

Telephone: +86-27-88041911

Fax: +86-27-88041911

E-mail: xy37138@163.com.

1. **Reporting of background should include**

**Problem definition:** compare the efficacy and safety of SAH and LRA for SAR

**Hypothesis statement:** SAH and LRA have similar effects and safety for SAR, but their effects are more outstanding in different aspects respectively

**Description of study outcome:** This meta-analysis suggested that SAH and LRA have similar effects and safety for SAR, but their effects are more outstanding in different aspects respectively. Meanwhile, the dose, duration and gender of patients may influence the anti-SAR effects of SAH and LRA.

**Type of exposure or intervention used:** Selective H1-antihistamine and Leukotriene Receptor antagonist;

**Type of study designs used:** Meta-analysis

**Study population:** patients with Seasonal allergic rhinitis took SAH or LRA

1. **Reporting of search strategy should include**

**Qualifications of searchers:** Jixiang Zhang and Yu Xu

**Search strategy, including time period include in the synthesis and keywords:**

PubMed from 1965 –May 2014

Cochrane Library from 1997 –May 2014

EMBASE from 1985 –May 2014

Selective H1-antihistamine; Leukotriene Receptor antagonist; Seasonal Allergic Rhinitis; Meta-analysis

**Effort to include all available studies, including contact with authors:** Yes

**Databases and registries searched:** PubMed, EMBASE, Cochrane Library

**Search software used, name and version, including special features used:** We did not employ any search software. EndNote was used to merge retrieved citations and eliminate duplications

**Use of hand searching:** Yes

**List of citations located and those excluded, including justification:** Figure 1 and Table 1

**Method of addressing articles published in languages other than English:** Translation software

**Method of handing abstracts and unpublished studies:** No unpublished studies were observed.

**Description of any contact with authors:** None

1. **Reporting of methods should include**

**Description of relevance or appropriateness of studies assembled for assessing the hypothesis to be tested:** Table 1

**Rationale for the selection and coding of data:** (1) randomized controlled trials or case-control studies; (2) compared the efficacy and safety of SAH with LRA for SAR; (3) had detailed information of cases and controls or could be calculated from the article text; (4) had specific evaluating standard.

**Documentation of how data were classified and coded:** Two investigators (Xu and Zhang) used a standard protocol and data-collection form. They discussed and then decide the data.

**Assessment of confounding:** No restricted for the analysis. Sensitivity analysis and subgroup analysis were used.

**Assessment of study quality, including binding of quality assessors; stratification or regression on possible predictors of study results:** The results of sensitivity analyses were very stable.

**Assessment of heterogeneity:** The chi-square-based *Q*-test and *I^2^* test

**Description of statistical methods in sufficient detail to be replicated:** A χ^2^-test-based Q statistic test was performed to assess the between-study heterogeneity. We also quantified the effect of heterogeneity by *I*^2^ test. When a significant Q test (*P*<0.05) or *I*^2^ >50% indicated heterogeneity across studies, the random effects model was used, or else the fixed effects model was used.

**Provision of appropriate tables and graphics:** We included the terms used for database search, 1 flow chart, 3 summary tables, 6 forest plots of all studies.

1. **Reporting of results should include**

**Graphic summarizing individual study estimates and overall estimate:** Table 2, Table 3, Figure 2, Figure 3, Figure 4, Figure 5, Figure 6, Figure 7

**Table giving descriptive information for each study included:** Table 2, Table 3

**Results of sensitivity testing:** Sensitivity analyses indicated that the results were stable.

**Indication of statistical uncertainty of findings:** 95% confidence intervals were presented with all summary estimates, *P* values and results of sensitivity analyses.

1. **Reporting of discussion should include**

**Quantitative assessment of bias:** According to the Egger’s Test, we found no evidence of publication bias for all analyses (Table2).

**Justification for exclusion:** We excluded studies: (1) did not compare the efficacy and safety of SAH with LRA; (2) not perennial allergic rhinitis; (3) case-only studies, case reports and review articles; (4) without sufficient information, and (5) without control group.

**Assessment of quality of included studies:** We discussed the results of the sensitivity analyses.

1. **Reporting of conclusions should include**

**Consideration of alternative explanations for observed results:** We discussed that potential unmeasured confounders such as differences of ethnicity, life style, environment background and other unknown factors may be the source of heterogeneity.

**Generalization of the conclusions:** This meta-analysis suggested that SAH and LRA have similar effects and safety for SAR, but their effects are more outstanding in different aspects respectively. Meanwhile, the dose, duration and gender of patients may influence the anti-SAR effects of SAH and LRA.

**Guidelines for future research:** Genic and environmental factors should be investigated in the future.

**Disclosure of funding source:** No funding supported this study.
